# The Molecular Basis for Remyelination Failure in Multiple Sclerosis

**DOI:** 10.3390/cells8080825

**Published:** 2019-08-03

**Authors:** Joel Gruchot, Vivien Weyers, Peter Göttle, Moritz Förster, Hans-Peter Hartung, Patrick Küry, David Kremer

**Affiliations:** Department of Neurology, Medical Faculty, Heinrich-Heine-University, 40225 Düsseldorf, Germany

**Keywords:** multiple sclerosis, remyelination, oligodendroglial precursor cells, neural stem cells, microglia

## Abstract

Myelin sheaths in the central nervous system (CNS) insulate axons and thereby allow saltatory nerve conduction, which is a prerequisite for complex brain function. Multiple sclerosis (MS), the most common inflammatory autoimmune disease of the CNS, leads to the destruction of myelin sheaths and the myelin-producing oligodendrocytes, thus leaving behind demyelinated axons prone to injury and degeneration. Clinically, this process manifests itself in significant neurological symptoms and disability. Resident oligodendroglial precursor cells (OPCs) and neural stem cells (NSCs) are present in the adult brain, and can differentiate into mature oligodendrocytes which then remyelinate the demyelinated axons. However, for multiple reasons, in MS the regenerative capacity of these cell populations diminishes significantly over time, ultimately leading to neurodegeneration, which currently remains untreatable. In addition, microglial cells, the resident innate immune cells of the CNS, can contribute further to inflammatory and degenerative axonal damage. Here, we review the molecular factors contributing to remyelination failure in MS by inhibiting OPC and NSC differentiation or modulating microglial behavior.

## 1. Introduction

Aside from multifocal inflammation and demyelination, neurodegeneration is one of the hallmarks of multiple sclerosis (MS). It represents the key factor driving clinical disability and the diminished quality of life in this commonest autoimmune disease of the central nervous system (CNS). The relapsing subtypes of MS (RMS) have become rather well treatable with a range of drugs. By contrast, progressive MS (PMS), characterized by the steady accumulation of neurological deficits and disability, remains a therapeutic challenge. Demyelination resulting from the autoimmune damage of oligodendrocytes and a loss of myelin sheaths lies at the core of MS. Myelin sheaths, which insulate axons and guarantee safe and reliable impulse propagation, are critical for normal neural function. Their loss leads to reduced axonal integrity, which over time, results in neuronal fallout, and consequently to fixed functional deficits. Partial replacement of lost oligodendrocytes and the (re)establishment of myelin sheaths around denuded axons can occur as a result of the activation, recruitment and differentiation of resident oligodendroglial precursor cells (OPCs) and neural stem cells (NSCs). These cells can differentiate and mature into myelin-sheath forming oligodendrocytes— a repair process commonly referred to as remyelination [1]. However, remyelination remains overall inefficient, which suggests that even though OPCs and NSCs are present in the MS brain, they are prevented from effectively differentiating into new myelin-producing cells by a variety of molecular mechanisms [2,3,4]. Of course, to what degree a reduced activity of one or the other immature cell population is the main factor for remyelination failure in MS, remains to be shown.

One of the most important cell types involved in these mechanisms are microglia, the resident innate immune cells of the CNS which modulate particularly OPC homeostasis, but also directly contribute to axonal degeneration. In this review, we investigate which factors influence OPC and NSC differentiation, with particular attention to the role of microglia in these processes (see Table S1).

## 2. Oligodendroglial Precursor Cells (OPCs)

Representing 5–8% of the total cell population of the adult brain, resident OPCs are found widely distributed throughout gray and white matter, and provide a source for myelin repair following CNS injury [5,6,7]. However, remyelination capacity remains overall inefficient and appears to decrease with age, despite the substantial number of OPCs found in demyelinated MS lesions. Interestingly, differences in the extent of myelin regeneration can be observed between individual lesions and patients, potentially indicating heterogeneity within the OPC population and the mechanisms underlying failing myelin repair in any given patient [8]. In this regard, recent publications point to a heterogeneity within the oligodendroglial cell population based on localization [9], origin [10] and additional lineage alterations upon demyelination [11]. This may explain different responses to demyelination and perhaps even a different susceptibility to age-associated functional decline [12]. To make things more complex, recently an additional contribution to myelin repair from partially lesioned oligodendrocytes has been suggested [13,14]. This is of particular interest, as surviving mature oligodendrocytes have classically been considered as passive bystanders of remyelination. However, using two different animal models, Duncan and colleagues have now demonstrated that mature oligodendrocytes extend processes towards demyelinated axons and ensheath them, while at the same time they are connected to surviving myelin sheaths. This may point to a so far unrecognized contribution of these cells to myelin repair. In summary, understanding the reasons for OPC differentiation and/or functional maturation failure in MS will hopefully help us to develop new strategies to improve remyelination. Accordingly, during the past years the focus of MS research has shifted to the identification of therapies that promote remyelination, for instance by modulating extrinsic and intrinsic factors that either act as the inhibitors or stimulators of OPC differentiation [15,16,17]. Furthermore, it was found that remyelination failure correlates with age and disease duration [18,19]. This is hypothesized to result from a reduced myelin debris clearance, and a decrease of factors secreted by monocytic cells promoting OPC differentiation [20]. Notably, aging also directly restricts oligodendroglial cell differentiation, resulting from age-related intrinsic changes in the mammalian target of the rapamycin (mTOR) signaling pathway, reducing differentiation which contributes to remyelination failure [21]. With regard to regional variations, it is known that gray and white matter lesions exhibit different capacities for remyelination. This finding is based on histochemical and electron microscopic studies in mostly chronic MS brains, which showed that the efficiency and degree of remyelination in cortical gray matter lesions (GML) is significantly higher than in white matter lesions (WML) [22]. These results may be explained by data demonstrating that the number of oligodendrocytes and OPCs is more than 6-fold higher in GML than in WML [23]. At least at first glance, this is somewhat surprising, as there is evidence that more oligodendrocytes and OPCs are present in normal appearing white matter (NAWM) than in normal appearing gray matter (NAGM) [24]. However, a rationale underlying this apparent paradox might be that in gray matter, oligodendroglial recruitment is simply more efficient [23]. Yet, other possible reasons for the differences in WML/GML remyelination capacity are being discussed, and range from the potential presence of different cortical and white matter oligodendrocyte and OPC subpopulations [25,26,27] to differences in microenvironment. In this regard, various factors could play a detrimental role regarding WML remyelination: The specific composition of the extracellular matrix (ECM) [28], a stronger inflammatory activity and microglial density [29], as well as an increased tendency to scar formation [23]. In addition, there also seems to be a relative overexpression of various inhibitors of OPC differentiation, including, inter alia, extracellular axonal ligands, such as PSA-NCAM [30], LINGO-1 [31], Jagged [32] and Galectin-4 [33], which were all shown to directly block OPC differentiation.

### Therapeutic Approaches to Promote OPC-Mediated Remyelination

Technically, one of the most effective approaches to neutralize potential inhibitors of OPC differentiation is targeted antibody therapy. An antibody that was already shown to have a good safety profile in phase 1 clinical trials for MS and amyotrophic lateral sclerosis (ALS) is ozanezumab (NCT01435993, NCT01424423). Ozanezumab is a humanized monoclonal antibody targeting the myelin-associated neurite outgrowth inhibitor NogoA. The histones of NogoA-expressing oligodendrocytes are highly acetylated, which is associated with an increased transcription of OPC differentiation inhibitors [34]. Clinically, the antibody-mediated neutralization of NogoA in a lysolecithin-induced animal model of experimental spinal cord demyelination results in enhanced remyelination and improved functional recovery [35]. Moreover, neutralization of NogoA might also be involved in enhanced synaptic plasticity and improved intrinsic repair in the adult CNS [36]. This is highly relevant, as none of the currently available MS treatments alleviates synaptic failure and network dysfunction [37]. Hence there is a need to further assess the therapeutic potential of NogoA neutralization.

Elezanumab is another humanized monoclonal antibody currently tested in phase 2 clinical trials for RMS (NCT03737851) and PMS (NCT03737812). This antibody is directed against the membrane-bound repulsive guidance molecule A (RGMa), which promotes inflammation and inhibits CNS regeneration and remyelination mainly via the multifunctional target receptor neogenin [38]. Anti-RGMa treatment leads to a decreased T cell proliferation, a decrease in pro-inflammatory interleukin production, a functional recovery in experimental autoimmune encephalomyelitis (EAE) [39] and to a prolonged conversion time to the secondary progressive disease phase [40].

Opicinumab, another monoclonal antibody, was also investigated in several clinical trials assessing its effectiveness and safety in MS patients. This antibody is directed against the Nogo-receptor interacting protein LINGO-1, which was found to negatively regulate OPC differentiation by activating RhoA and inhibiting the Akt signaling pathways [41]. The phase 2 SYNERGY trial (NCT01864148) investigated the safety and efficacy of opicinumab as an add-on therapy to intramuscular interferon beta-1a in patients with RMS. Although the primary outcome (a multicomponent improvement of function over 72 weeks) was not met, some trends emerged in subgroup analyses: Younger RMS patients with shorter disease duration and magnetic resonance imaging (MRI) features suggestive of more preserved brain tissue (i.e., bigger whole-brain and thalamic volume at baseline) had a greater therapy effect. The RENEW trial (NCT01721161) testing opicinumab in patients with acute optic neuritis (AON) did not show a significant difference in the intent-to-treat population (ITT), either. However, it could show a possible beneficial effect of opicinumab on remyelination in a pre-specified per-protocol analysis after 32 weeks of treatment, with the recovery of P100 latencies of full-field visual evoked potential (FF-VEP; [42]) as a primary outcome.

Finally, the humanized anti-pHERV-W Env antibody temelimab yielded promising results in a recently completed phase 2b study in RMS (NCT02782858) after a previous phase 2a study had already yielded encouraging results [43]. Temelimab is directed against the pathogenic envelope protein (Env) of a human endogenous retrovirus (HERV) belonging to the HERV-W family. Although the primary MRI-related endpoints of the study, with an emphasis on inflammation in the trial period from week 12 to 24, were not met, a significant dose-dependent beneficial effect on brain and thalamic atrophy, as well as on the number of T1 hypointense “black holes” could be shown in the secondary endpoints in the trial period from week 24 to 48. In addition, a potential benefit on remyelination as indicated by Magnetization Transfer Ratio (MTR) was observed. A more recent phase I study has now investigated higher doses of temelimab, and could confirm the previously shown good safety profile (NCT03574428). These results will certainly lead to further clinical studies focused on PMS. In addition to these emerging antibodies, research simultaneously focuses on the repurposing of well-established drugs regarding possible neuroregenerative effects. Accordingly, there are currently studies underway investigating simvastatin (NCT03387670), quetiapine (NCT02087631) and clemastine fumarate (NCT02040298). Simvastatin is a 3-hydroxy-3-methyl-glutaryl-coenzyme A (HMG-CoA) reductase inhibitor commonly used for the treatment of hypercholesterolemia. Regarding its effect on remyelination there are contradictory results. While a number of studies have found that simvastatin stimulates OPC survival and differentiation [44,45], others have suggested the opposite [46]. Quetiapin on the other hand, is an atypical neuroleptic drug, which has been reported to increase OPC differentiation and myelin protein production [47]. Finally, clemastine is a histamine H1 receptor blocker, which was shown to increase OPC differentiation in disease entities such as hypoxic brain injury [48,49].

## 3. Neural Stem Cells (NSCs)

In the past years, growing interest has focused on endogenous and transplanted neural stem cells (NSCs) and their potential to provide oligodendrocyte replacement and to mediate myelin repair. NSCs are multipotent cells which can self-renew and differentiate into neurons and glial cells [50,51,52], most notably, under appropriate circumstances, also into oligodendroglia. In the adult mammalian brain, at least two distinct niches, the subventricular zone (SVZ) of the lateral ventricle, and the subgranular zone (SGZ) of the dentate gyrus, are described to harbor NSCs. In contrast to the SVZ, SGZ-derived stem cells mainly differentiate into neurons unless they are genetically reprogrammed or trophically manipulated to produce oligodendrocytes [53,54,55,56,57,58,59,60]. In this regard, during the last years, several factors were identified which can instruct and drive oligodendrogenesis from NSCs. More importantly, it was demonstrated that SVZ NSC-derived oligodendroglial cells can contribute significantly to remyelination in several demyelination mouse models [61,62,63,64,65]. Of note, in some of these studies, more NSC-derived than OPC-derived newly generated oligodendrocytes were detected [64,65], pointing to the relevance of these cells regarding CNS repair. As these studies identified NSCs as a major contributing source for myelin repair, the question arises why NSC-mediated remyelination in MS is overall inefficient and even decreases over time. In this regard, several potential explanations emerged during the past years.

### 3.1. Aging

As already mentioned above, aging could play a role in failing remyelination. In this context it was shown that the aged SVZ suffers from a reduced number of ventricle-contacting astrocytes. These cells are the precursors for so-called transit-amplifying cells (TAPs), which are highly proliferative, and can give rise to newly differentiated oligodendrocytes. This is in line with the recent suggestion that human cellular senescence is responsible for a diminished remyelination potential in progressive MS patients [66]. Moreover, it has been repeatedly shown that in the aged brain less neurogenesis occurs [67,68,69,70], which has been explained by increased cell cycle lengths, a lower availability of growth factors and an accumulation of inhibitory factors. It is therefore conceivable that similar mechanisms apply to gliogenesis, i.e., the production of new oligodendrocytes. However, so far corresponding research has yielded contradicting results. While it was shown that neurosphere-mediated oligodendrogenesis in response to growth factors declines with age [68], several other groups found that NSC-derived oligodendrogenesis remains constant [69,70,71]. While an age-related decrease in trophic factor expression was described in the human SVZ by some research groups [72,73], Weissleder and colleagues reported an increase or stable expression in transcripts encoding trophic factors or their receptors, such as epidermal growth factor (EGF), fibroblast growth factor 2 (FGF2) and Erb-B2 receptor tyrosine kinase 4 (ErbB4; [70]). On the other hand, measurements of ^14^C incorporation into genomic DNA (a result of nuclear tests during the Cold War) demonstrated that newly generated oligodendrocytes can still be detected around the lateral wall of the adult human SVZ [74], thereby providing evidence for ongoing oligodendrogenesis in the adult.

### 3.2. Inflammation

In the mouse MS model, EAE inflammation was demonstrated to modulate NSC differentiation [75,76,77], and autoantibodies in EAE showed a strong affinity to SVZ NSCs [78]. EAE was also found to induce spontaneous apoptosis of neural stem and progenitor cells (NPCs) in vitro [76]. 

While several EAE studies provide evidence that NSCs can modulate chemokine levels, resulting in an impaired recruitment of immune cells to the CNS [79,80], these signaling molecules are also important for the attraction of endogenous NSCs into white matter tracts [75]. Of note, one EAE study showed that chitinase 3-like-3 (Chi3l3) activates the epidermal growth factor receptor (EGFR) on neural stem cells, which results in decreased disease severity [81]. Moreover, chemokines are important for stem cell proliferation, resulting in a lower capacity to differentiate into oligodendrocytes, and an increased differentiation into neuronal progenitors [77,82]. In contrast, transplanted NSCs were shown to participate directly in the remyelination of damaged axons [83], but also to influence OPC-based myelin regeneration after cuprizone-mediated demyelination [84]. A recent study using single cell transcriptomics on aging neural stem cell niches provided evidence of increased T cell infiltration in aged animals, which apparently impairs NSC proliferation via interferon-γ secretion [85].

### 3.3. Factors Involved in NSC-Based Oligodendrogenesis

A number of different factors and signaling pathways which modulate NSC-derived oligodendrogenesis have been described (summarized in [86]). The majority of the encoded proteins exert similar roles on both OPCs and NSCs. However, a few transcriptional/epigenetic regulators, such as Gli1 [87], Sirt1 [88], nuclear factor I X (NFIX; [89]), B-cell leukemia homeodomain 1 (Pbx1, [90]), prospero-related homeobox 1 gene (Prox1, [91]), drosha and nuclear factor IB (NFIB, [60]), exhibit stem cell-specific effects, and might therefore be of particular interest regarding alternative myelin repair pathways. For example, it could be shown that the negative regulatory effect on remyelination by Gli1 and Sirt1 could be partially reversed by using small molecule inhibitors (GANT61 for Gli1 and EX-527 for Sirt1, respectively). Furthermore, it was shown that nuclear factor-erythroid 2-related factor 2 (NRF2) leads to impaired differentiation and proliferation rates of hippocampal NSCs [92]. This is an interesting observation, given the fact that dimethylfumarate (DMF), a well-established and potent oral MS medication, is known to enhance NRF2 expression. Finally, FGFR3 activation was recently shown to redirect the differentiation of SVZ-derived NSCs into oligodendrocytes promoting remyelination [93]. Another point that should be considered regarding NSC-based remyelination is the heterogeneity within NSC niches. While signaling pathways involving Wnt/β-catenin, Prox1, Olig2, and Sox10 are enriched in the dorsal microdomain of the SVZ [94,95,96,97], Pbx1 can be detected throughout the entire niche [62]. p57kip2, on the other hand, appears to dominate in the lateral wall of the SVZ [86]. In contrast, NSCs located in the innermost parts of the granule cell layer of the hilus mainly give rise to neurons, but can also form new oligodendrocytes following overexpression of achaete-scute family bHLH transcription factor 1 (Ascl1, [54,58]) or the inhibition of factors such as Prox1, [97], neurofibromatosis type I (Nf1, [59]), Drosha [60], ubiquitin-specific peptidase 9, X-linked (Usp9x, [98]) or p57kip2 [55].

## 4. Microglia

OPC and NSC homeostasis cannot be seen in isolation from other cell populations or physiological processes. In this regard, the innate immune system, i.e., resident microglia and peripherally-derived infiltrating macrophages, have been shown to be essential for remyelination. This includes axon regeneration, the clearance of myelin debris and the release of neurotrophic factors that promote OPC differentiation [99]. Microglia are the resident innate immune cells of the CNS and play an important role in the MS disease process. On the one hand, they can adopt a pro-inflammatory phenotype, classically known as M1, contributing to inflammation and axonal damage. On the other hand, they can also take on a restorative phenotype, known as M2, which is associated with anti-inflammation, tissue repair and phagocytosis of debris. This process, classically referred to as polarization, and as of late challenged in the scientific community [100], lies at the core of the complex role of microglia in de- and remyelination [101].

### 4.1. Phagocytosis of Myelin Debris

Phagocytosis of myelin debris in the MS brain is essential for the initiation of neuro-repair as myelin debris inhibits OPC differentiation and, by doing so, remyelination [102]. Among others, Fractalkine receptor (CX3CR1), which is expressed at high levels on microglia, has been identified to exert an impact on microglial phagocytic capacity. CX3CR1-deficient mice treated with cuprizone were shown to have a reduced microglial phagocytic capacity, leading to a persistent presence of myelin debris, which results in inefficient remyelination due to impaired OPC recruitment [103]. Another factor relevant for phagocytosis is triggering receptor expressed on myeloid cells (TREM2). Long-term studies of TREM2 knockout mice showed impaired myelin debris clearance, axonal dystrophy, oligodendrocyte reduction, and persistent demyelination after prolonged cuprizone treatment [104]. In addition, in the inflammatory setting of EAE, a blockade of TREM2 leads to disease progression, resulting in diffuse demyelination [105]. Other players affecting phagocytosis are the TAM family receptors MerTK and Axl. Axl knockout mice subjected to MOG-EAE feature a decreased number of activated microglia in lesions resulting in reduced myelin debris clearance [106]. Regarding MerTK, in vitro studies in human microglia which were stimulated with TGF-β revealed a strong increase in myelin ingestion based on an upregulation of MerTK and its ligands Protein S and growth arrest specific 6 (GAS6) in comparison to controls [107]. Further studies on monocyte-derived macrophages from MS patients then identified the MerTK pathway as being essential for myelin phagocytosis. MS macrophages displayed a reduced myelin uptake capacity, correlating with lower levels of MerTK and its ligands compared to healthy controls [108]. In addition to the above-mentioned factors, a recently published study has identified the pHERV-W envelope protein Env as a stimulator of microglia-associated inflammation in MS. Env reduces microglial phagocytic capacity by downregulating TREM2 and MerTK resulting in decreased myelin debris clearance. Moreover, Env was found to drive microglia-mediated axonal damage resulting in the leakage of myelin and intra-axonal indicative of axonal degeneration [109].

### 4.2. Microglial Stimulation of OPC Differentiation

Besides inefficient myelin debris clearance, failing paracrine stimulation of OPC differentiation by microglia is another key factor contributing to inefficient remyelination in MS. In this context, it was discovered that fibronectin aggregates accumulate in the extracellular matrix (EZM) during chronic demyelination processes and impede remyelination via inhibiting oligodendrocyte differentiation. Matrix metalloproteinases (MMPs) on the other hand, can modulate the EZM and are able to split fibronectin—as it is the case for MMP7. Microglia were identified as a major source of the MMP7 proenzyme proMMP7, which is reduced in chronic active and inactive MS lesions correlating with higher amounts of fibronectin aggregates disrupting OPC differentiation capacity [110]. In general, both M1 and M2 phenotypes are simultaneously present in MS lesions, but for successful OPC differentiation and remyelination a switch from M1 to M2 seems to be essential [111]. In this regard, the long noncoding RNA (lncRNA) GAS5 was shown to regulate microglial polarization with increased levels being found in activated ameboid microglia in MS brains. GAS5 apparently averts M2 polarization by suppressing the transcription of TRF4, via a recruitment of the polycomb repressive complex 2 (PRC2). In EAE, interference with GAS5 in microglia attenuates disease progression and promotes remyelination [112]. Beyond that, microglia are able to promote remyelination via a complex repertoire of secreted chemokines and cytokines which stimulate oligodendroglial differentiation [113]. Factors that have been identified as being supportive in this regard are, for instance, CXCL12 [114,115,116], semaphorin 3F [117], Activin-A [111] and Galectin-3 [118]. It is noteworthy that in chronic persistent inflammation, as it occurs in MS, microglia secrete many of these supporting factors at reduced levels, thereby contributing to ineffective remyelination [17,111,119].

## 5. Discussion

While the past years have seen the approval of highly potent immunomodulatory drugs which led to a paradigm shift in the treatment of RMS, therapies that stimulate myelin repair and prevent neurodegeneration are still unavailable. However, even though ultimately rather ineffective, the brain’s own repair mechanisms provide us with helpful leads as to where to start the search for such therapies. Of course, in order to claim credible relevance for MS, pre-clinical studies which aim to identify promising molecules have to be conducted in a context as close as possible to the human paradigm, i.e., in human cells and/or in human tissue, which are, of course, difficult to procure. Nonetheless, the quest for myelin repair enhancement in MS has already prompted the initiation of several trials assessing the clinical effectiveness of molecules known to inhibit or stimulate OPC differentiation [16]. Of note, some of these molecules are not exclusively studied for MS, but also in patients with other CNS diseases, such as amyotrophic lateral sclerosis (ALS) or spinal cord injury (SCI) as these disorders share certain aspects of MS pathophysiology. Interestingly and probably due to their proximity to mature myelin-forming oligodendrocytes, clinical research has so far exclusively focused on molecules stimulating the differentiation of OPCs. In contrast, factors facilitating NSC differentiation or beneficially modulating microglia have not attracted an equal level of attention. This is somewhat surprising, as in some experimental animal studies more NSC-derived than OPC-derived newly generated oligodendrocytes were detected. In addition, so far there has not been a single trial investigating molecules aimed at stimulating microglia-associated phagocytosis. Yet, the plain fact that a completely new generation of potential MS medications is currently being studied remains reason enough for cautious optimism. Of note, regarding the molecular mechanisms of such therapeutic agents, there are some important caveats. First of all, potential therapeutic molecules must be cell-specific, and should not pleiotropically interfere with physiologically required pathways. As they have to work on cell populations inside the CNS, they must also be able to cross the blood-brain-barrier (BBB). While the BBB is leaky during acute MS relapses, as demonstrated by MRI-based Gadolinium enhancement, it is, to a large degree, re-established afterwards. However, microglia-mediated neurodegeneration is continuing even behind a closed BBB. In order to solve this problem, among other approaches virus-based CNS drug delivery systems have been suggested which are able to transport therapeutic molecules across an intact BBB [120]. Of course, in order to maximize tropism for (oligodendro)glia, viral systems based on JC polyomavirus (JCPyV) seem to be the most promising (Chao et al., 2018). This leads us to the next challenge, which consists in finding the right therapeutic “window of opportunity”. An application of regenerative agents too late during the disease course could be fruitless, as axons might have already degenerated irreversibly. In summary, it is very likely that the MS therapy of the future will consist of a two-pronged approach, uniting established immunomodulatory treatments with remyelinating ones. Despite the aforementioned complex issues, it is very encouraging to observe the new developments in the emerging field of clinical remyelination therapy. Hopefully, we will soon have new therapeutic options at our disposal that will enable us to effectively treat both the inflammatory and neurodegenerative aspects of MS, in order not only to prevent new damage, but also to restore lost function.

## Supplementary Materials

The following are available online at https://www.mdpi.com/2073-4409/8/8/825/s1, Table S1. Molecules and their potential impact on remyelination in the central nervous system (CNS).
