# Supplementary material for: The Molecular Basis for Remyelination Failure in Multiple Sclerosis
Source: Cells. 2019 Aug 3;8(8):825. doi: 10.3390/cells8080825 (PMC6721708; doi:10.3390/cells8080825)
Supplement: Supplementary file 1 [file cells-08-00825-s001.pdf]

Cell type	Molecule	Potential impact on remyelination
Oligodendroglial Precursor Cells (OPCs)	Myelin-associated neurite outgrowth inhibitor NogoA	Impairing remyelination<br>Impeding neurite outgrowth<br>Enhanced remyelination by anti-NogoA-Ab ozanezumab
	Membrane-bound repulsive guidance molecule A (RGMa)	Impairing remyelination<br>Modulates T cell response<br>Impeding neurite outgrowth<br>Enhanced remyelination by anti-RGMa-Ab elezanumab
	Nogo-receptor interacting protein LINGO-1	Impairing remyelination<br>Impeding OPC differentiation<br>Enhanced remyelination by anti-LINGO-1-Ab opicinumab
	Envelope protein (Env) of the pathogenic human endogenous retrovirus type W (pHERV-W)	Impairing remyelination<br>Impeding OPC differentiation<br>Enhanced remyelination and reduced neurodegeneration by anti-pHERV-W Env-Ab temelimab
	Simvastatin	(Potentially) stimulating OPC differentiation and survival
	Quetiapine	Stimulating OPC differentiation and myelin protein production
	Clemastine fumarate	Stimulating OPC differentiation and remyelination
Neural Stem Cells (NSCs)	Chitinase 3-like-3 (Chi3l3)	Activating epidermal growth factor receptor (EGFR)<br>Inducing pro-oligodendrogenic transcription factor signature<br>Enhancing oligodendrogenesis
	Gli1	Impairing remyelination<br>Immobilizing and impeding the differentiation of resident NSCs<br>Enhanced remyelination by GANT61 (SMI of Gli1)
	Sirt1	Impairing remyelination<br>Suppressing oligodendrogenesis from NSCs<br>Activating downstream Akt and p38 MAPK<br>Limiting the expansion of SVZ NSCs and OPCs<br>Enhanced oligodendrogenesis by EX-527 (SMI of Sirt1)
	nuclear factor I X (NFIX)	Impairing remyelination<br>Suppressing oligodendrogenesis from NSCs
	B-cell leukemia homeodomain 1 (Pbx1)	Early regulator of SVZ neuro- und oligodendrogenesis<br>Priming factor to activate neuron-specific genes Dcx and Th
	prospero-related homeobox 1 gene (Prox1)	Enhancing SGZ neurogenesis and neuronal differentiation<br>Downstream target of β-catenin-TCF/LEF signaling
	drosha and nuclear factor IB (NFIB)	Enhancing oligodendrogenesis<br>Drosha enhances SGZ neurogenesis and suppresses oligodendrogenesis via suppression of NFIB
	nuclear factor-erythroid 2-related factor 2 (NRF2)	Enhancing SGZ neurogenesis<br>Enhancing neuronal differentiation<br>Regulating the expression of several antioxidant enzymes
	fibroblast growth factor receptor-3 (FGFR3)	Promoting remyelination<br>Enhancing SVZ oligodendrogenesis<br>Promoting migration of SVZ-derived OPCs to more distal areas
Microglia and peripherally-derived	Fractalkine receptor (CX3CR1)	Promoting remyelination<br>Increasing microglial phagocytic capacity<br>Improving extracellular myelin debris clearance
	Triggering receptor expressed on myeloid cells (TREM2)	Promoting remyelination<br>Increasing microglial phagocytic capacity<br>Improving extracellular myelin debris clearance

	TAM family receptors MerTK and Axl	Promoting remyelination<br>Activating microglia and increasing microglial phagocytic capacity<br>Improving extracellular myelin debris clearance
	Envelope protein (Env) of the pathogenic human endogenous retrovirus type W (pHERV-W)	Impairing remyelination<br>Stimulating microglia-associated inflammation and neurodegeneration<br>Decreasing microglial phagocytic capacity
	Matrix metalloproteinase 7 (MMP7)	Promoting remyelination<br>Splitting fibronectin aggregates that disrupt OPC differentiation<br>Secreted by microglia
	Long noncoding RNA (lncRNA) GAS5	Impairing remyelination<br>Averting microglial M2 polarization by suppressing TRF4
	CXCL12	Promoting OPC differentiation<br>Secreted by microglia
	Semaphorin 3F	Promoting OPC differentiation<br>Improving OPC recruitment<br>Secreted by microglia
	Activin-A	Promoting OPC differentiation<br>Downstream activation of Rac-Cdc42 GTPases, Akt and mTOR<br>Secreted by microglia
	Galectin-3	Promoting OPC differentiation<br>Improving myelin integrity and function<br>Secreted by microglia

Table S1. Molecules and their potential impact on remyelination in the central nervous system (CNS). Ab=antibody; SMI=small molecule inhibitor; SVZ=subventricular zone; SGZ=subgranular zone; MAPK=Mitogen-activated protein kinases; TAM= TYRO3, Axl and MerTK family of receptor tyrosine kinases (RTKs).
